# Death from Chloroform

**Published:** 1871-01

**Authors:** 


Death from Chloroform.—O. K. Grant, of Brunswick, Me., about 40 years of age, and of late a clerk in the Census Bureau in Washington, died suddenly Monday, from the effects of chloroform, which had been administered by Dr. ——, one of the ablest surgeons in that town, preparatory to an examination.

